# Assessment of pMDI inhaler technique in children and adolescents with cystic fibrosis

**DOI:** 10.1016/j.jped.2026.101624

**Published:** 2026-09-30

**Authors:** Ana Maria Coelho Bastos, Marcos Vinicius da Silva Pone, Patrícia Fernandes Barreto Machado Costa, Margareth Catoia Varela, Saint Clair dos Santos Gomes Junior

**Affiliations:** a Fundação Oswaldo Cruz (Fiocruz), Instituto Nacional de Saúde da Mulher, da Criança e do Adolescente Fernandes Figueira, Postgraduate Program in Applied Research in Child and Women's Health, Rio de Janeiro, RJ, Brazil; b Fundação Oswaldo Cruz (Fiocruz), Instituto Nacional de Saúde da Mulher, da Criança e do Adolescente Fernandes Figueira, Rio de Janeiro, RJ, Brazil; c Fundação Oswaldo Cruz (Fiocruz), Instituto Nacional de Infectologia Evandro Chagas, Health Surveillance and Immunization Research Unit (LIVS), Rio de Janeiro, RJ, Brazil

**Keywords:** Cystic fibrosis, Medication adherence, Child, Adolescent, Inhaler technique

## Abstract

**Objective:**

To assess the quality of pressurized metered-dose inhaler (pMDI) technique among patients with cystic fibrosis (CF) and their caregivers, and identify associated clinical and socioeconomic factors.

**Methods:**

Cross-sectional study of 146 CF patients (≤ 18 years) followed at a reference center in 2025. Inhaler technique was assessed using a study-specific 11-step checklist with age-based adaptations. Adequate technique required correct performance of ≥ 70% of eligible steps, including all essential steps. Socioeconomic and clinical data were obtained from medical records and structured interviews.

**Results:**

The overall adequacy rate was 68.4%. The 0–5-year age group showed higher adequacy than those ≥ 6 years (85.1% vs. 60.6%). Main errors were re-shaking for the second puff in children < 6 years (31.9%) and slow exhalation in those ≥ 6 years (81.8%). Multivariate logistic regression identified early childhood (0–5 years, OR = 5.16; 95% CI: 1.78–14.91; p = 0.002) and family income of 2–3 minimum wages (OR = 0.30; 95% CI: 0.10–0.90; p = 0.032) as independent predictors of technique quality.

**Conclusions:**

Nearly one-third of children and adolescents with CF had inadequate pMDI technique (31.6%). Respiratory coordination and re-shaking for the second puff were the main errors identified. Early childhood (0–5 years) was independently associated with better technique, whereas family income of 2–3 minimum wages predicted poorer performance. The developed checklist proved viable for multidisciplinary CF consultations. Longitudinal, multicenter studies using validated instruments are needed to establish causality and assess the impact of systematic educational interventions.

## Introduction

Cystic fibrosis (CF) is a chronic, progressive, multisystemic genetic disorder caused by mutations in the CFTR (Cystic Fibrosis Transmembrane Conductance Regulator) gene [1]. CFTR dysfunction disrupts ionic balance across epithelial surfaces, leading to chronic pulmonary disease and multiorgan dysfunction [1,2].

Pulmonary disease is the leading cause of morbidity and mortality in CF patients. Treatment encompasses multiple inhaled medications — bronchodilators, mucolytics, antibiotics, and anti-inflammatory agents — delivered via different devices, along with daily physiotherapy, enzyme replacement, nutritional support, and psychological care, totaling up to three hours of daily treatment [3].

Inhaled therapy enables targeted drug deposition in the airways with rapid onset of action and a lower risk of systemic effects, and the pressurized metered-dose inhaler (pMDI) is one of the oldest and most widely prescribed devices worldwide. However, therapeutic effectiveness depends critically on the quality of the technique employed, and the literature consistently reports a high frequency of errors across all age groups, including healthcare professionals [4,5]. Although several instruments for assessing inhaler technique exist, most were developed for obstructive diseases such as asthma and Chronic Obstructive Pulmonary Disease (COPD), and none has undergone full validation for clinical or research use [6].

This challenge is particularly pronounced in CF, where the complexity of the therapeutic regimen requires patients and their caregivers to simultaneously master multiple devices [7,8]. Adherence to inhaled therapies — already recognized as lower than that observed with other routes of administration [7,9] — is associated with longer hospitalizations, frequent pulmonary exacerbations, and decline in lung function [8,10]. Although studies addressing pMDI use in CF exist, detailed assessments of inhaler technique in this population, particularly in children and adolescents, remain scarce. Therefore, the present study aims to evaluate pMDI inhaler technique in pediatric patients with CF and their caregivers at a tertiary reference center.

## Methods

This was a cross-sectional observational study conducted with pediatric CF patients (≤ 18 years) followed at a CF Reference Center in 2025 and 2026. The study was approved by the Institutional Review Board. Written informed consent was obtained from all participants' legal guardians, and age-appropriate assent was obtained from participants aged 6–18 years.

### Eligibility criteria

Inclusion criteria were confirmed CF diagnosis, regular follow-up (≥ 3 pneumology visits in the preceding year), age ≤ 18 years, and current pMDI use. Patients with congenital craniofacial anomalies precluding correct device use or with tracheostomy were excluded.

### Inhaler technique assessment instrument

A study-specific checklist was developed based on the most highly rated protocols identified by the systematic reviews of De Vos et al. [6] and Rodríguez-Martínez et al. [11], specifically those of Welch [12], Sleath [13], Kamps [14], Boccuti [15], Zambelli [16] and Manzella [17], as well as GINA (Global Initiative for Asthma) 2025 [18] and GOLD 2024 [19] guidelines. The instrument comprises 11 steps: (1) cap removal; (2) inhaler-to-spacer connection; (3) mask-to-spacer connection; (4) inhaler shaking*; (5) vertical positioning; (6) slow complete exhalation; (7) mask fit or mouthpiece positioning between lips and teeth; (8) inhaler actuation; (9) slow deep inhalation*; (10) 10-second inspiratory breath-hold*; (11) re-shaking for second puff. (*Essential steps.)

For children under 6 years, steps requiring forced respiratory control were replaced by the tidal breathing technique (10 respiratory cycles or 20-second count after actuation), per GINA 2025 recommendations [18]. In this age group, the technique was necessarily performed by the caregiver, given the child's limited respiratory and motor coordination to execute the maneuver independently. For patients aged 6 years and older, self-administration was expected; however, reports of whether the technique was performed independently or with caregiver supervision were inconsistent, with some families describing variable practice (occasionally independent, occasionally supervised), and therefore could not be reliably classified for analysis. Technique was considered adequate when participants correctly performed ≥ 70% of eligible steps, including all essential steps mandatorily. The 70% cut-off was established by team consensus as a minimum operational proficiency criterion, given the variability in the number of eligible steps across participants and the absence of an internationally validated threshold. This approach sought to balance the practicality of a linear scoring system with the rigor of instruments that prioritize critical steps, such as those of Kamps et al. [14] and Boccuti et al. [15] Assessment was performed with placebo pMDI devices (same model as the patient's own device). All identified technique errors were corrected at the end of the assessment for ethical reasons.

Instruction in pMDI use was not provided as part of the study protocol or in temporal proximity to the assessment; rather, it followed the routine standard of care at the studied institution, in which patients receive ongoing guidance from the multidisciplinary team, including respiratory physiotherapists and pediatric pulmonologists, during outpatient follow-up visits.

### Data collection

Socioeconomic variables included: age, sex, municipality of origin, area of residence (rural/urban), family monthly income in minimum wages (MW), number of household members, government benefit receipt, and caregiver educational level. Family monthly income was expressed in multiples of the Brazilian national minimum wage (salário mínimo), a fixed value set annually by the federal government, corresponding to R$1,518.00 (approximately US$271, based on the 2025 average exchange rate) at the time of data collection. Clinical variables included: nutritional status (WHO 2006/2007 z-score) [20], forced expiratory volume in one second (FEV1, % predicted), hospital admissions in the past 24 months, treatment regimen complexity (> 3 inhaled medications/day = complex), sputum bacteriology, and CFTR modulator use. FEV1 was categorized as: normal/mild (≥ 70%), moderate (40–69%) or severe (< 40%). No formal sample size calculation was performed; the study included all eligible patients followed at the center during the data collection period, constituting a convenience sample.

### Statistical analysis

Descriptive analysis expressed categorical variables as absolute and relative frequencies, and continuous variables as median and interquartile range (IQR), given non-parametric distribution confirmed by the Shapiro-Wilk test. Bivariate analysis was performed to identify factors associated with technique inadequacy (p < 0.20 threshold or clinical relevance for multivariate inclusion). Multivariable logistic regression with backward elimination was applied (minimizing the Akaike Information Criterion). Association strength was estimated by adjusted Odds Ratio (95% CI), with significance at *p* < 0.05. Analyses were performed in JASP version 0.19.1; data management was carried out on REDCap (Research Electronic Data Capture). Missing data were minimal and handled by complete case analysis.

## Results

Of the 196 patients followed at the center between 2025 and 2026, 50 were excluded: 33 were absent during the data collection period, 13 were not using a pMDI, 3 had guardians who declined participation, and 1 did not meet the minimum visit requirement. The final sample comprised 146 participants.

### Sample characteristics

The sample comprised 51.4% female participants, with a median age of 8.0 years (IQR: 4.0–12.0). All were from Rio de Janeiro State (41.8% from the capital). The majority resided in urban areas (84.2%). Most caregivers had completed secondary education or higher (58.9%); 23.3% had not completed primary schooling. Family income was ≤ 1 MW in 43.2% of households. Key socioeconomic characteristics are summarized in Table 1.

**Table 1:** Socioeconomic characteristics of participants (n = 146).

Variable	n (%)
Age group	
0 to 5 years	47 (32.2%)
6 to 11 years	56 (38.4%)
12 to 18 years	43 (29.5%)
Female sex	75 (51.4%)
Origin – Rio de Janeiro (capital)	61 (41.8%)
Urban area of residence	123 (84.2%)
Family income	
≤ 1 MW	63 (43.2%)
> 1 and < 2 MW	22 (15.1%)
2–3 MW	23 (15.8%)
> 3 MW	32 (21.9%)
Government benefit recipient	75 (51.4%)
Caregiver: secondary education or higher	86 (58.9%)
Medication administered by patient themselves	42 (28.8%)

MW, minimum wage.

Regarding nutritional status, the majority of participants (88.9%) were classified as eutrophic, at risk of overweight, or overweight, while 6.2% were obese and 4.8% were underweight or severely underweight. Among the 99 patients who performed spirometry, mean FEV1 was 88.6% (±21.83); only 3.0% had FEV1 < 40%. *Burkholderia Cepacia Complex* (BCC) colonization was identified in 2.1%, and 53.4% were receiving CFTR modulators.

### Error frequency by checklist step

The under-6 group showed a lower overall error frequency than the ≥ 6-year group (14.8% vs. 39.3%). In the younger group, the most frequent error was incomplete cycle repetition for a second puff (31.9%), followed by inhaler shaking (8.5%). In participants aged ≥ 6 years, the highest error rates were observed for slow complete exhalation (81.8%) and mouthpiece positioning between the lips and teeth (53.1%). Essential steps of slow deep inhalation and inspiratory breath-hold showed error frequencies of 27.3% and 22.2%, respectively. Detailed step-by-step frequencies are presented in Table 2, Table 3, which present technique steps and error frequencies separately for children under 6 years, for whom the technique was performed by the caregiver, and for those aged 6 years and older, for whom self-administration was expected but could not be reliably classified due to inconsistent reporting of caregiver involvement.

**Table 2:** Checklist step-by-step error frequency – participants aged < 6 years (n = 47).

Step	Correct n (%)	Incorrect n (%)
1. Cap removal	47 (100.0%)	0 (0.0%)
2. Inhaler-to-spacer connection	47 (100.0%)	0 (0.0%)
3. Mask-to-spacer connection	47 (100.0%)	0 (0.0%)
4. Inhaler shaking*	43 (91.5%)	4 (8.5%)
5. Vertical positioning	47 (100.0%)	0 (0.0%)
6. Mask fit on face	47 (100.0%)	0 (0.0%)
7. Inhaler actuation	47 (100.0%)	0 (0.0%)
8. 20 seconds or 10 tidal breaths*	43 (91.5%)	4 (8.5%)
9. Cycle repetition for 2nd puff	32 (68.1%)	15 (31.9%)
Overall adequacy	40 (85.1%)	7 (14.8%)

⁎ Essential steps.

**Table 3:** Checklist step-by-step error frequency – participants aged ≥ 6 years (n = 99).

Step	Correct n (%)	Incorrect n (%)
1. Cap removal	99 (100.0%)	0 (0.0%)
2. Inhaler-to-spacer connection (n = 78)	77 (98.7%)	1 (0.01%)
3. Inhaler shaking*	88 (88.9%)	11 (11.1%)
4. Vertical positioning	98 (99.0%)	1 (1.0%)
5. Slow complete exhalation	18 (18.2%)	81 (81.8%)
6. Mouthpiece positioning between lips/teeth (n = 64)	30 (46.8%)	34 (53.1%)
7. Mask fit on face (n = 35)	35 (100.0%)	0 (0.0%)
8. Inhaler actuation	99 (100.0%)	0 (0.0%)
9. Slow deep inhalation*	72 (72.7%)	27 (27.3%)
10. 10-second inspiratory breath-hold*	77 (77.8%)	22 (22.2%)
11. Cycle repetition for 2nd puff	87 (87.9%)	12 (12.1%)
Overall adequacy	60 (60.6%)	39 (39.3%)

⁎ Essential steps.

### Bivariate analysis

The overall technique adequacy rate was 68.4% (100/146). Variables with *p* < 0.20 in the bivariate analysis were: age group (*p* = 0.003), area of residence (*p* = 0.169), family income (*p* = 0.077), government benefit receipt (*p* = 0.099) and CFTR modulator use (*p* = 0.114). No clinical variable reached statistical significance. Key bivariate results are shown in Table 4.

**Table 4:** Multivariate logistic regression model for predictors of adequate pMDI technique in Cystic Fibrosis patients.

Predictor variable	OR	95% CI	p-value
Age group			
0–5 years	5.16	1.78 – 14.91	0.002
≥ 6 years (ref.)	1.00	—	—
Urban area of residence	2.48	0.74 – 8.28	0.140
Rural area (ref.)	1.00	—	—
Family income			
> 3 MW	0.82	0.29 – 2.34	0.716
2–3 MW	0.30	0.10 – 0.90	0.032
> 1 and < 2 MW	1.54	0.43 – 5.52	0.506
≤ 1 MW (ref.)	1.00	—	—

OR, Odds Ratio; CI, Confidence Interval; MW, minimum wage.

Model adjusted by backward elimination strategy. Variables removed: government benefits receipt, CFTR modulator use, nutritional status and lung function. Bivariate analysis showed an overall technique adequacy rate of 68.4%: age group 0–5 years — adequate 40 (40.0%) vs. inadequate 7 (15.2%), p = 0.003; family income 2–3 MW — adequate 11 (11.0%) vs. inadequate 12 (26.1%), p = 0.077.

### Multivariable logistic regression

The final model (AIC = 155.9; Tjur's R² = 0.144) identified the 0–5-year age group and family income of 2–3 MW as independent predictors of technique quality. Children in early childhood were 5.16 times more likely to perform the technique correctly than older age groups (OR = 5.16; 95% CI: 1.78–14.91; *p* = 0.002). Families with income between 2 and 3 MW showed significantly lower odds of adequate technique compared to those with income ≤ 1 MW (OR = 0.30; 95% CI: 0.10–0.90; *p* = 0.032). Area of residence was retained for model adjustment but did not reach significance (*p* = 0.140). Full model results are presented in Table 4.

## Discussion

This study documented an overall pMDI technique adequacy rate of 68.4% in a pediatric CF center, a result comparable to error rates of 24–31% reported in large studies of patients with obstructive diseases such as asthma and COPD [4,21]. It must be noted, however, that the CF treatment burden is substantially greater, involving multiple inhaled device classes used simultaneously, which may compromise individual device technique [3,7].

MDI-delivered medications in CF are limited primarily to bronchodilators and, to a lesser extent, inhaled corticosteroids; other therapies, such as mucolytics and most inhaled antibiotics, are delivered only by nebulization, and caution should be taken when extrapolating these findings to this broader spectrum of inhaled CF treatments. Nonetheless, bronchodilators remain the most commonly prescribed medication class in CF, used extensively across this population [2]. The pMDI technique errors identified in this study are therefore clinically relevant to the majority of CF patients.

The analysis of checklist errors revealed a pattern consistent with previous reports by Sanchis et al. [4] and Plaza et al.: [5] failures concentrate in respiratory coordination and control steps. Among participants ≥ 6 years, slow complete exhalation was the most neglected step (81.8%), with most participants reporting unawareness of its requirement. Unlike many checklists that simply instruct "exhale normally" or "exhale completely," slow exhalation is specifically recommended prior to pMDI actuation, as it promotes the slow, coordinated inhalation these devices require [22,23].

The second most frequent error was incorrect mouthpiece placement between the lips and teeth (53.1%), which promotes oropharyngeal deposition and reduces pulmonary bioavailability [24]. In the preschool group, the main failure involved incomplete cycle repetition for a second puff, including re-shaking (31.9%), consistent with findings of a 2019 systematic review reporting this error in 40–45% of patients [25].

The strongest predictor of technique adequacy in the multivariate analysis was the 0–5-year age group (OR = 5.16; *p* = 0.002). This result aligns with CF adherence literature demonstrating better performance in younger children due to active caregiver involvement [8,9,26,27]. In this sample, caregivers performed the technique in 100% of under-6-year participants. Additionally, the technique recommended for this age group — mask, spacer and tidal breathing [18] — is inherently simpler, as it eliminates forced respiratory maneuvers, which represent the primary error sources in older patients [4]. This reduced technical complexity likely contributes to the higher adequacy observed in younger children, independent of caregiver involvement alone.

Family income emerged as the second independent predictor. The reduced odds of adequate technique in the 2–3 MW stratum compared to those with ≤ 1 MW (OR = 0.30; *p* = 0.032) suggest that intermediate-income families face distinct challenges from both lower- and higher-income households. A plausible explanation is that these families often fall just above the eligibility threshold for government assistance programs targeted at lower-income groups — such as the Continuous Cash Benefit (BPC) and Bolsa Família, both restricted to households with per capita income below one-quarter of the minimum wage — yet still face substantial indirect costs of CF care, including transportation, supplies, and caregiver income loss [28,29]. Pinto et al. [28] estimated a median annual non-medical cost of R$2,156.56 for CF families in Rio de Janeiro, with over 100% income loss post-diagnosis and 69% of families resorting to loans; Alves and Bueno [29] similarly found that most caregivers of pediatric CF patients faced difficulty acquiring medications and were unable to work outside the home. Thus, while lower-income families are more likely to access social protection networks, intermediate-income households face a coverage gap that may compromise home-based treatment, including inhaled therapy. This pattern parallels Seyoum et al. [30], who found that even among CF families with formal health insurance in the United States, 64% reported disease-related financial burden — reinforcing that income and insurance coverage alone do not eliminate the indirect costs of chronic disease.

The absence of association between clinical variables and technique quality likely reflects the current mild clinical profile of the cohort, where severe disease markers were infrequent: only 4.8% were classified as underweight or severely underweight, 3.0% had FEV1 < 40%, and 2.1% had BCC colonization. This profile might be partly attributable to the high proportion of patients using CFTR modulators (53.4%), medications associated with significant clinical improvement in patients with CF [2].

The study-specific checklist proved viable for multidisciplinary CF consultations, with direct observation and immediate error correction also serving as an educational intervention, as recommended by GINA [18]. Although not formally timed, checklist application required less than 1 minute, with total encounter time (including error correction) averaging approximately 5 minutes, supporting its feasibility for routine clinical use.

Limitations include: (1) cross-sectional design, precluding causal inference; (2) absence of formal validation of the checklist instrument, and reliability testing precluded by single-evaluator assessment, introducing potential measurement error and limiting comparability with other studies, although item selection was grounded in protocols rated highly by systematic reviews and current guidelines; (3) single-center convenience sample, restricting generalizability, with certain subgroups, such as rural residence (n = 18), yielding wide confidence intervals in the multivariate model; (4) underrepresentation of patients with severe disease, which may have limited the detection of associations with clinical variables; (5) potential observer-induced performance bias (Hawthorne effect), whereby participants may have performed better than usual under observation; (6) the 70% adequacy threshold was established by team consensus rather than derived from a validated correlation with clinical or pulmonary deposition outcomes, reflecting the broader absence of a standardized scoring benchmark across the pMDI technique literature.

Nearly one-third of the children and adolescents with CF followed at the reference center demonstrated inadequate pMDI technique (31.6%). Respiratory coordination steps — prior exhalation and inspiratory breath-hold — and cycle repetition for the second puff represent priority targets for individualized educational interventions. Early childhood (0–5 years) was independently associated with better pMDI technique, whereas a family income of 2–3 minimum wages predicted poorer performance compared to the lowest income group, emphasizing the need for focused education in these at-risk populations. The developed checklist proved to be a viable clinical tool for multidisciplinary CF consultations. Further longitudinal, multicenter studies employing validated instruments are warranted to establish causality of the identified associations and to assess the sustained impact of systematic educational interventions on inhaler technique in patients with CF.

## Funding

This study was financed in part by the Coordenação de Aperfeiçoamento de Pessoal de Nível Superior – Brasil (CAPES) – Finance Code 001.

## Ethics approval

Approved by the Institutional Review Board. Informed consent was obtained from all participants and/or their legal guardians.

## Declaration of generative AI

During the preparation of this work, the author used Claude (Anthropic) to assist with manuscript structuring, formatting adaptation to journal guidelines, and language editing. The author reviewed and edited the content as needed and takes full responsibility for the content of the published article.

## Data availability

The datasets generated and analyzed during the current study are not publicly available due to patient privacy considerations but are available from the corresponding author upon reasonable request.

## Conflicts of interest

The authors declare no conflicts of interest.
